# Transmission of environmentally responsible behavior between tourist destination employees and tourists: The role of moral elevation and environmental knowledge

**DOI:** 10.3389/fpsyg.2022.1027736

**Published:** 2022-11-30

**Authors:** Weijiao Ye, Ziqiang Li, Yuyan Xu

**Affiliations:** ^1^College of Business Administration, Capital University of Economics and Business, Beijing, China; ^2^College of Economics and Management, Huazhong Agricultural University, Wuhan, Hubei, China; ^3^Anxi College of Tea Science, Fujian Agriculture and Forestry University, Fujian, China

**Keywords:** tourists’ environmentally responsible behavior, employees’ environmentally responsible behavior, moral elevation, environmental knowledge, behavioral contagion

## Abstract

**Introduction:**

Tourists’ environmental misconduct is the primary reason for the environmental destruction that tourist sites experience; nevertheless, their environmentally responsible behavior is also a major push for the improvement of the environment. The main goal of this study is to induce tourists to adopt proactive environmental responsibility behaviors.

**Methods:**

A total of 455 valid questionnaires were obtained from China and analyzed using multiple linear regression.

**Results:**

The findings of this study indicate employees’ environmentally responsible behavior (E-ERB) in tourist destinations has a positive impact on tourists’ environmentally responsible behavior (T-ERB). In the mediating variable of moral elevation, the correlation between E-ERB and T-ERB is mediated by elevating emotions and views of humanity. And desire to be a better person did not play a mediating role in the relationship between E-ERB and T-ERB. Additionally, environmental knowledge moderates the transmission path of the impact of E-ERB and T-ERB *via* elevating emotions. With high environmental knowledge, the transmission path of the impact of employees’ environmentally responsible behavior of the tourist destination on tourists’ environmentally responsible behavior *via* elevating emotions will be enhanced.

**Discussion:**

We propose a new perspective to explain the transmission mechanism between employees’ environmentally responsible behavior and tourists’ environmentally responsible behavior in tourism destinations, which will help to expand our understanding of the relationship between employees’ behavior and tourists’ behavior. We expect our study to spark more exploration of the contagion of positive behavior in the field of environmental psychology.

## Introduction

Damage to the environment and a heightened understanding of sustainable development have prompted a greater emphasis on environmental protection in tourist destinations. Long-term, the deterioration of the environment in tourism destinations not only impedes the sustainability of the tourism industry but also poses a significant threat to the tourism industry’s long-term viability (Confente and Scarpi, 2021). Thus, many studies have focused on individual positive environmental behaviors such as pro-environmental behavior (Wang et al., 2020; Shah et al., 2021), environmentally friendly behavior (Ren et al., 2021), and environmentally responsible behavior (ERB; Li Q. et al., 2020; Saleem et al., 2021). Environmentally responsible behavior (ERB) is given greater importance since it is related to corporate social responsibility in that both emphasize the consideration of stakeholders’ interests while gratifying self-image (Li S. et al., 2020). ERB focuses on the social responsibility of employees, residents, and tourists to the environment of tourist destinations, and promotes the sustainable use of environmental resources in tourist destinations. ERB includes a range of activities such as compliance with destination rules, waste reduction, recycling, and participation in environmental conservation projects (Wu et al., 2021).

In the previous decades of ERB research, the theory of planned behavior (Saleem et al., 2021), norm activation theory (Confente and Scarpi, 2021; Wu et al., 2021), and stimulus-organism-response framework have been the most prominent theoretical frameworks (Lyon et al., 2018). These theoretical frameworks have enriched the theoretical explanations of ERB antecedents, but they have failed to investigate the transmission mechanisms between individual behaviors. Several studies in recent years have begun to focus on inter-individual versus inter-group behavioral transfer. McDonald and Crandall (2015) contend that group circumstances create social norms that restrain individual behavior. Examples like social norms for environmental protection are produced by group conditions that suppress individuals’ environmentally destructive behavior. Liu et al. (2019) used the broken windows theory to explain the mechanism by which an individual’s environmentally destructive behavior triggers a group’s environmentally destructive behavior. Examples include tourists’ perceptions of the environmental quality of tourist destinations affecting tourists’ ERB. Previous studies are important for elucidating the transmission of individual and group behaviors, but there is a dearth of research on motivating individuals to actively engage in environmentally beneficial behaviors. From the standpoint of emotional contagion theory, Gilal et al. (2020) explained the process by which teachers’ environmental responsibility is conveyed to students *via* emotional contagion. We believe there is another explanation for behavior transmission between individuals. Haidt (2000) developed elevation based on Fredrickson’s (2000) broaden-and-build model, which claims that when a person witnesses unexpected acts of kindness, friendliness, and compassion in another person, he/she feels elevated and motivated to help others and improve himself/herself. The majority of academics agree with Haidt’s perspective (Monroe, 2020; Shulman et al., 2021). According to Haidt’s moral viewpoint, our work examines the transmission mechanism of E-ERB and T-ERB.

The primary subjects of ERB’s antecedent research are employees and inhabitants of tourist destinations, as well as tourists. The antecedents of ERB differ amongst subjects. Tourism destination employees’ ERB (T-ERB) is influenced mainly by tourism firms’ human resource management practices and sustainable development strategies (Roche et al., 2016). Residents’ ERB is primarily influenced by perceptions of sustainability (Lin and Lee, 2022), place dependence, perceived benefits (Lee and Oh, 2018), resident’s perceived of justice (Liu et al., 2022), and community attachment (Safshekan et al., 2020). Awareness of tourism’s negative consequences (Confente and Scarpi, 2021), positive emotions (Chen and Huang, 2022), subjective norms and attitudes toward ERB (Saleem et al., 2021), and tourists’ awareness of consequences and tourists’ ascribed responsibility have the greatest influence on tourists’ ERB (Wu et al., 2021). While the antecedents of tourists’ ERB (T-ERB) have been extensively studied, the influence of travel destination employees on tourist behavior has been disregarded. Despite the notion that moral norm theory may adequately explain the ERB of tourists to limit behavior detrimental to environmental preservation by the group moral norm, it cannot induce the ERB of tourists. Therefore, we use Haidt’s (2000) notion of moral elevation as a “black box” to explain the relationship between employees’ ERB and tourists’ ERB.

Furthermore, environmental knowledge is based on personal views of environmental issues and a broad understanding of the implementation, concepts, and relationships of the natural environment and major ecosystems (Fryxell and Lo, 2003). We believe environmental knowledge influences the moral elevation that E-ERB has on tourists. For example, the more environmental knowledge tourists possess, the greater their awareness of the influence of E-ERB on the destination’s environment, and consequently their moral elevation. The environmental knowledge of tourists reflects not only their grasp of the current state of the environment and the impact of human activities on the environment but also their concern for environmental changes and the impact of their daily actions on the environment. Thus, we broaden the settings of ERB research by using environmental knowledge as a moderating variable for E-ERB to influence T-ERB *via* moral elevation.

This study investigates the transmission mechanism of the correlation between E-ERB and T-ERB in tourism destinations, using moral elevation as a mediating variable and environmental knowledge as a moderating variable. Our study has three goals: first, to investigate the correlation between E-ERB and T-ERB in tourism destinations. Second, to investigate the moral elevation mechanism that mediates the correlation between E-ERB and T-ERB. Third, examine the moderating effect of environmental knowledge on the influence of E-ERB on the moral elevation of tourists. Our study makes three major theoretical contributions: first, our study proposes a novel theoretical explanatory framework for research aimed at inducing tourists’ ERB, explaining the relationship between employee ERB and tourist ERB in tourism destinations, and examining the impact of employee conduct on tourists’ behavior. Second, we use moral variables as a mediator between employees’ behavior and tourists’ behavior, so enhancing our understanding of ERB’s internal mechanism. Third, our study extends the study of ERB boundary conditions by incorporating environmental knowledge as a moderating variable between E-ERB and tourists’ moral elevation, which facilitates the understanding of how individuals’ environmental knowledge affects moral elevation and thus promotes their ERB through tourists’ perceptions of E-ERB.

## Literature review and hypotheses

### E-ERB and T-ERB

Behavioral contagion is the propensity and process whereby an individual’s behavior induces the same behavior in a close relative. Behavioral contagion is a sort of social contagion that highlights that when a person’s behavior acts as a stimulus, his or her neighbors will exhibit the same behavior, hence establishing the phenomena of behavioral transmission. Gustave Le Bon was the first to propose the possibility of behavioral contagion, suggesting that implication is the primary cause of behavioral contagion. Some scholars have proposed that social standards limit the likelihood of harmful behavior spreading. Wheeler (1966) stated that four fundamental criteria must exist for the behavioral contagion process to take place: (a) Individual X witnesses Individual Y’s behavior B. (b) Behavior B has an agonistic stimulus impact on Individual X. (c) no hurdles or inhibitors are preventing Individual X from engaging in Behavior B. (d) Individual X is not pretending. Additionally, Wheeler argued that behavioral contagion occurs not only in the domain of socially unacceptable acts but also in other behavioral domains. Numerous researchers have discovered the occurrence of behavioral contagion in a variety of behavioral domains, including consumer behavior contagion, new technology adoption behavior, and financial behavior contagion (Du and Kamakura, 2011; Langley et al., 2012; Yuan et al., 2022). Haidt discovered in his early studies on aversion that people’s antipathy to unpleasant things is symptomatic of their desire for and veneration of good things. Haidt conducted numerous experiments to validate his theories. Haidt (2000) found in his research that when people witness unexpected acts of kindness, they report feeling shocked, affected, and experiencing feelings of love and beauty. Participants’ descriptions of their feelings about moral behavior in the Haidt experiment indicated that the cognitive structures of the participants impacted their perspectives of humans and stimulated more prosocial aspirations. Specifically, moral beauty stimulates a moral emotion of complimenting others, a moral emotion that makes individuals feel good about human society and desire moral beauty. Haidt defines the feeling of participants in his experiment as a sense of moral elevation. Moral elevation can serve as a “moral reset button” in the human psyche and is propelled by moral exemplars and virtuous actions (Haidt, 2003).

Environmentally responsible behavior entails both conscious and unconscious activities performed by humans to lessen environmental issues and even improve the environment (Lee et al., 2013). E-ERB is the proactive and voluntary involvement of employees in environmentally responsible behavior that is not part of their job responsibilities. Due to their work, employees may be more concerned with environmental issues, leading them to conduct environmentally responsible acts, both consciously and unconsciously, to lessen environmental problems or improve the environment. T-ERB refers to tourists’ environmentally responsible behavior, which is the deliberate and unconscious actions tourists do to decrease environmental problems or enhance the environment during their travels. At a time when the United Nations is emphasizing environmental damage as a major threat to human life, social norms are encouraging people to reduce environmental pollution. Environmental protection research has also begun to incorporate moral factors into the ERB study framework; for example, Confente and Scarpi (2021) found that moral obligation or moral ideology positively influences behavioral intentions toward environmental responsibility. Social norms posit that when conserving the environment becomes a group’s social standard, people reduce their environmentally destructive conduct to avoid exclusion or loss of social standing. However, social norms do not imply that people will take proactive measures to save the environment. Consequently, taking the effort to safeguard the environment is still a moral act that would be warm and motivating according to the existing societal norms. According to Wheeler’s (1966) four necessary criteria for behavioral contagion, a) tourists witness employees’ ERB. b) ERBs provide an agonistic stimulus for tourists because, in the sense of desire and admiration for high moral traits, tourists feel positive and pleasant when they witness E-ERB and intentionally gravitate toward these behaviors. c) There are no barriers and inhibitors that hinder tourists’ ERB, and d) tourists are not required to fake ERB because proactive ERB is not a socially obligatory behavior. The study’s hypothesis is therefore proposed.

*H1*: E-ERB has a positive impact on T-ERB.

### The mediating role of moral elevation

In an experiment conducted by Haidt (2000), participants were asked to record instances in which they witnessed someone exhibiting greater or better human qualities. According to the results of the experiment, the participants felt motivated and warmed when recalling these moral behaviors. Moreover, these participants expressed a desire to emulate these moral role models to become better people. In moral psychological analysis, moral elevation consists of a succession of reactions (Aquino et al., 2011). First, moral elevation induces a distinct sensation of warmth and expansion in the chest, followed by feelings of love, admiration, and affection for the person who caused it. The research of Algoe and Haidt (2009) indicated that moral elevation encompasses both emotional and bodily sensations, such as emotions of motivation or elation and a lump in the throat or warmth in the chest. Second, witnessing moral behavior will change people’s outlook on the world by making them view it more optimistically. Similar to other moral feelings, moral elevation can be a key source of moral motivation to inspire virtue or immediately deter an individual from engaging in immoral behavior (Lai et al., 2014; Shulman et al., 2021). For instance, those who experience moral elevation are more likely to help others and pursue more ethically significant life goals (Van de Vyver and Abrams, 2015). Finally, desire to be a better person. Many scholars identify the desire to be a better person and to be more receptive to others as reasons connected with moral elevation (Silvers and Haidt, 2008). Thus, moral elevation is measured in terms of elevating emotions, views of humanity, and the desire to be a better person.

Scholars have noted that ERB can reflect individuals’ concerns and attitudes, ecological knowledge, and commitment to environmental issues (Safshekan et al., 2020). As stated previously, employees’ ERB in tourist destinations is regarded as a voluntary act performed by individuals outside of their employment responsibilities, as tourist destinations typically have dedicated employees responsible for preserving the scenic environment at specified times. Thus, when employees at a tourist destination take the initiative to pick up trash thrown on the ground by tourists or clean up graffiti by tourists on the spot, tourists perceive it as an unexpectedly ethical act (because they could have done otherwise), and evoke a positive mood. We believe that when tourists observe employees engaging in environmentally responsible behavior, they will perceive that environmental protection is not a matter of indifference and that someone is taking the initiative to protect the environment. And employees’ environmental protection behavior is not influenced by self-interest, as these activities are neither demanded nor rewarded in their work. The results of an experiment in which Haidt (2000) had some participants view uplifting video clips and a control group view other videos revealed that participants who viewed the uplifting video clips reported feeling more loving and inspired and had a greater desire to engage in pro-social and affiliative actions. Similarly, other researchers have demonstrated that moral beauty increases this main effect of moral elevation from different backgrounds, samples of different ages and ethnicities, and using a variety of manipulation types (e.g., Algoe and Haidt, 2009; Cox, 2010). Empirical research also supports the idea proposed by Haidt that people will induce moral elevation whenever they are exposed to positive moral behavior (e.g., Algoe and Haidt, 2009; Aquino et al., 2011). The study’s hypothesis is therefore proposed.

*H2*: E-ERB has a positive impact on moral elevation (a. elevating emotions; b. views of humanity; c. desire to be a better person).

Similar to other emotions, moral elevation is caused by a triggering incident that causes physical changes in a person and prompts some form of action propensity (Ding et al., 2018). According to Haidt (2003), moral elevation is indicated when it is related to the desire to imitate moral role models, so that moral elevation would lead to the adoption of more pro-social activities, such as donating to charity. Freeman et al. (2009) proved that exposing individuals to morally virtuous behaviors increases their generosity. Thus, moral elevation is regarded as a crucial factor in a person’s prosocial behavior (Schnall et al., 2010). Just as anger may lead to uncontrollable violence, positive emotional experiences motivate people to do things that are good for others. Erickson et al. (2018) proposed that as an emotion that is theorized as a contraction of the ego, moral elevation increases spiritual transcendence (Van Cappellen et al., 2013) and reduces prejudice (Lai et al., 2014). Aquino and McFerran. (2011) interpret positive behavior in terms of components of the moral elevation experience that initiate action tendencies such as the desire to emulate moral role models and pro-social actions. Previous research has also confirmed that ethical elevation is associated with a range of pro-social behavioral outcomes, such as volunteering (Cox, 2010), organizational citizenship behavior in the workplace (Aquino et al., 2011), reduced racial bias (Freeman et al., 2009), and increased financial giving (Aquino et al., 2011). Furthermore, moral elevation is believed to inspire people to emulate moral role models. Schnall et al. (2010) organized participants in their study to watch a moral elevation video, and subsequently, 70% of the participants were willing to participate in this experiment without compensation. Rullo et al. (2022) confirmed that witnessing unusual moral behavior can elicit feelings of moral elevation that further translate into pro-social intentions and behaviors. Thus, tourists engage in ERB when they are emotionally affected, inspired to change their view of humanity, and motivated to better themselves. Consequently, the hypothesis is as follows:

*H3*: Moral elevation (a. elevating emotions; b. views of humanity; c. desire to be a better person) has a positive impact on T-ERB.

*H4*: Moral elevation (a. elevating emotions; b. views of humanity; c. desire to be a better person) mediates the relationship between E-ERB and T-ERB.

### The moderating role of environmental knowledge

Knowledge is seen as the total of human knowledge and experience accumulated during the period required to create the world. Environmental knowledge indicates an individual’s awareness of environmental issues and general knowledge of facts, concepts, and relationships about the natural environment and major ecosystems (Fryxell and Lo, 2003). It is a well-established truth that knowledge is acquired through cognitive activity and that the level of an individual’s cognitive development is determined by their existing knowledge. Specifically, an individual’s existing environmental knowledge reflects their level of environmental consciousness. The cognitive-behavioral theory proposes that cognition plays a mediating and moderating role in emotion and behavior (Merrill and Strauman, 2004). Thus, an individual’s environmental knowledge is very closely related to his emotions and behavior. Gifford and Nilsson (2014) suggest that if a person is ignorant of environmental issues, he or she is unlikely to be consciously concerned about them or to take some conscious action to benefit the environment (Liu et al., 2020). Many researchers have also argued that it is impossible to make informed environmental decisions if one lacks or possesses insufficient environmental knowledge (Levine and Strube, 2012). Thus, environmental knowledge is believed to contribute to environmental attitudes and behaviors (Kollmuss and Agyeman, 2002; Lin and Niu, 2018). For example, consumers’ awareness of the need of using products made from recyclable plastics for environmental conservation and their willingness to purchase such products increase proportionally with their environmental literacy.

**Figure 1:**
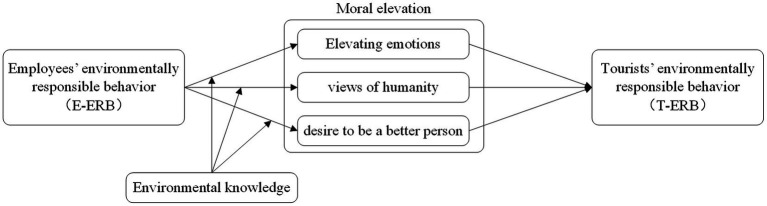
Research model.

Environmental knowledge is also considered to be the information that a person possesses about the interrelationship between people and the environment. This knowledge reveals how individuals perceive their responsibility for the environment, which leads to their environmental behaviors. That is, individuals are also aware of how their environmental behaviors contribute to sustainable development (Fan et al., 2012). Ogbeide et al. (2015) discovered that consumers with extensive environmental knowledge were more willing to spend money on environmentally friendly products. Crandall et al. (2013) indicated that cognitive rigidity and low educational attainment are factors that contribute to “prejudiced personality.” The low interest in environmental protection topics in countries with low education levels may also mean that they are not educated about the environment and therefore do not realize that being proactive in protecting the environment is as meaningful as being proactive in helping others. Moral elevation is a warm emotion evoked by witnessing virtue or acts of moral virtue accompanied by a desire to be imitated (Monroe, 2020). The idea of this study is that tourists with more environmental knowledge are more likely to admire and be moved by E-ERB and have the desire to emulate it than those with less environmental knowledge. Because they know that engaging in proactive environmentally responsible behavior is not a universal phenomenon, but an altruistic act. And people know the significance of doing so for the environment and the development of human society. Haryanto’s (2014) research proposes a positive correlation between increased environmental knowledge and increased environmental attitudes and behaviors, which are beneficial to the environment. Fryxell and Lo (2003) considered environmental knowledge as a major variable in explaining individual pro-environmental behavior. Studies by Amoah and Addoah (2021), Liu et al. (2020), and Xie and Lu (2022) also confirmed the important role of environmental knowledge on pro-environmental behavior. Accordingly, the hypothesis was formulated. And the research model for this study was established (Figure 1).

*H5*: The environmental knowledge moderates the indirect effects of E-ERB on T-ERB via moral elevation (a. elevating emotions; b. views of humanity; c. desire to be a better person).

## Materials and methods

### Sample and procedure

This study collected data *via* a questionnaire survey. The majority of the samples were collected from three forest tourist destinations in Quanzhou, Fujian Province, China. There are two primary ways of data gathering, one of which is to rely on the personnel of the scenic area to urge tourists to fill out a survey after the tour. The second section is completed by the researcher at the end of the tourist attraction while the tourists wait for their companions. The first portion of data was collected between 06-15-2022 and 07-15-2022, while the second portion was collected between 06-15-2022 and 06-20-2022. In particular, duplicates of the same questionnaire were delivered twice (rather than two parts of one questionnaire). To prevent the questionnaire results from diverging from the tourists’ actual conduct, since environmentally responsible behavior is a socially laudable behavior. Using a procedural control strategy, we minimized this response bias during the survey process. First, before the survey, participants were told of this survey’s purpose and requirements to reduce their anxiety. Participants were notified that the survey was conducted anonymously. Besides, we created an environment in which no one was present throughout the completion of the questionnaire so that participants could be comfortable that no one would know their genuine responses. And all questionnaires were completed by the tourists themselves.

The employees in tourist destinations handed a total of 450 surveys to tourists. We collected a total of 372 valid questionnaires, with a response rate of 82.7%, after eliminating the invalid questionnaires with incomplete information. The researcher received a total of 100 questionnaires for distribution to tourists. After eliminating invalid surveys with inadequate information, 83 valid questionnaires were gathered, yielding an 83% response rate. For this research, a total of 455 questionnaires were gathered. Among them, 54.5% are male and 45.4% are female; the majority are between the ages of 19–30 and 31–45, comprising 38.3 and 40.4%, respectively; and the majority have a Bachelor’s degree or above, including 23.9 and 31.4%, respectively. 88.4% of the population’s income falls between 3000 and 15000RMB. The majority of ecotourism visits fall between 2 and 4.

### Measures

Before the official research, 50 questionnaires were selected and sent to tourists of a scenic destination for this study’s preliminary research, and the questionnaires were changed based on the questionnaire returns and the comments of experts. Using a seven-point Likert scale, all questions were evaluated (1 = strongly disagree, 7 = strongly agree). Both T-ERB and E-ERB used the 6-item scale developed by He et al. (2018) based on Cheng et al. (2013) and Chiu et al. (2014). The representative E-ERB items are “Employees of tourist destinations take the initiative to stop others from being destructive to the environment of (name of destination)” and “When employees of tourist destinations saw garbage and tree branches on the ground, he/she will pick them up and put them in the trash.” The Cronbach’s *α* in this paper is 0.836. T-ERB representative items are “I try to convince others to protect the natural environment at (name of destination)” and “If there are environmental clean-up activities at (name of destination), I would be willing to attend,” with Cronbach’s *α* 0.780. Moral elevation was measured using the scale adopted by Zheng et al. (2019). The scale consists of 3 sub-dimensions, including elevating emotions (3 items), views of humanity (4 items), and desire to be a better person (4 items). The representative question of elevating emotions is “The proactive behaviors of the tourist destination’s employees to protect the environment makes me feel moved.” The Cronbach’s *α* in this research is 0.922. The representative question of views of humanity is “The proactive behaviors of the tourist destination’s employees to protect the environment makes me believe that there is still some good in the world.” The Cronbach’s *α* in this research is 0.926. The representative question of desire to be a better person is “The proactive behaviors of the tourist destination’s employees to protect the environment makes me desire to be a better person.” The Cronbach’s *α* in this research is 0.934. Environmental knowledge was measured using a 7-item scale developed by Liu et al. (2020) based on Ajzen (1991), with representative questions such as “Carbon dioxide contributes to the creation of the greenhouse effect” and “The increasing carbon dioxide in the atmosphere is one of the factors causing a warming climate,” The Cronbach’s *α* in this research is 0.836. The measuring items are available in the Appendix 1.

In addition, by Bernerth and Aguinis’ (2016) guidelines for controlling factors, we included as control variables gender, age, education, income, and the number of trips, which are theoretically connected with ERB. The gender codes are 0 for females and 1 for males. The age is coded as follows: 1 = 18 or below, 2 = 19–30, 3 = 31–45, 4 = 46–60, 5 = 61 or above. The education is coded as follows: 1 = Secondary school or below, 2 = high school, 3 = college, 4 = Bachelor, 5 = Master or above. Income is coded as follows: 1 = 3000 RMB or below, 2 = 3001–5000 RMB, 3 = 5001–10000 RMB, 4 = 10000-15000RMB, 5 = 15000 or above. Besides, the frequency of travel is the phrase “How often have you visited ecotourism attractions?” Tourists can complete the form according to their cases.

## Results

### Common method biases and confirmatory factor analyses

In this study, one-period data and tourist-reported information were utilized. As proposed by Podsakoff et al. (2003), we used Harman’s one-factor test for common method bias to determine the validity of the sample variables. Six factors have characteristic roots larger than 1 according to unrotated principal component analysis. Moreover, the first component explained 18.11%<40% of the variance, whereas the total variance was explained by 60.86%. Consequently, the model does not have a single factor that explains the great majority of the variance and is not constrained by common method bias.

This study examined the discriminant validity of E-ERB, elevating emotions, views of humanity, desire to be a better person, environmental knowledge, and T-ERB using confirmatory factor analysis (CFA). The results are presented in Table 1, and the fit of the six-factor model is higher than other models: *χ*^2^ = 638.237, df = 455, *χ*^2^/df = 1.403, CFI = 0.973, GFI = 0.923, TLI = 0.970, RMSEA = 0.030. Therefore, the six variables E-ERB, elevating emotions, views of humanity, the desire to be a better person, environmental knowledge, and T-ERB had discriminant validity in this study. Besides, the six variables’ average variance extracted is>0.5 with E-ERB (0.504), elevating emotions (0.866), views of humanity (0.819), desire to be a better person(0.834), environmental knowledge (0.506), and T-ERB (0.619). And each variables composite reliability is>0.7 with E-ERB (0.877), elevating emotions (0.951), views of humanity (0.948), desire to be a better person (0.953), environmental knowledge (0.878), and T-ERB (0.919). These analyses demonstrate that the data in this investigation are reliable and valid, providing sufficient support for testing hypotheses.

**Table 1:** Confirmatory factor analysis.

Models	*χ* ^2^	df	*χ*^2^/df	CFI	GFI	TLI	RMSEA
6-Factor model	638.237	455	1.403	0.973	0.923	0.970	0.030
5-Factor model^e^	1596.509	457	3.493	0.831	0.817	0.745	0.074
4-Factor model^d^	3378.968	459	7.362	0.568	0.638	0.533	0.118
3-Factor model^c^	4402.975	461	9.551	0.416	0.588	0.372	0.137
2-Factor model^b^	4992.806	463	10.784	0.329	0.531	0.281	0.147
1-Factor model^a^	5513.370	464	14.616	0.252	0.252	0.201	0.155

*N* = 455. ^a^E-ERB + Elevating emotions + Views of humanity + Environmental knowledge + Desire to be a better person + T-ERB. ^b^E-ERB + Elevating emotions + Views of humanity + Environmental knowledge + Desire to be a better person, T-ERB. ^c^E-ERB + Elevating emotions + Views of humanity + Desire to be a better person, Environmental knowledge, T-ERB. ^d^E-ERB + Views of humanity + Elevating emotions, Desire to be a better person, Environmental knowledge, T-ERB. ^e^E-ERB + Elevating emotions, Views of humanity, Desire to be a better person, Environmental knowledge, T-ERB.

### Descriptive statistics and correlations

Table 2 summarizes the means, standard deviations, and correlations of the variables used in this study. We used Spearman’s coefficient to analyze the correlations as our study had dichotomous variables (e.g., gender). E-ERB is positively correlated with elevating emotions (*r* = 0.102, *p* < 0.05), views of humanity (*r* = 0.158, *p* < 0.01), desire to be a better person (*r* = 0.26, *p* < 0.05) and T-ERB (*r* = 0.323, *p* < 0.01) were significantly positively correlated. Elevating emotions were positively correlated with T-ERB (*r* = 0.157, *p* < 0.01). The views of humanity were positively correlated with T-ERB (*r* = 0.213, *p* < 0.01). The desire to be a better person was positively correlated with T-ERB (*r* = 0.132, *p* < 0.01). These results provide preliminary evidence for the subsequent analysis.

**Table 2:** Means, standard deviations, and correlations of all variables in the study.

Variables	1	2	3	4	5	6	7	8	9	10	11
1. Gender	–										
2. Age	0.115^*^	–									
3. Education	−0.004	0.032	–								
4. Income	−0.022	−0.057	0.045	–							
5. Frequency of travel	−0.048	0.023	0.059	−0.001	–						
6. E-ERB	−0.077	0.045	0.112^*^	0.066	0.139^**^	(0.836)					
7. Elevating emotions	0.021	0.002	0.039	−0.014	−0.061	0.102^*^	(0.922)				
8. Views of humanity	0.011	0.011	0.238^**^	0.121^**^	0.116^*^	0.158^**^	−0.032	(0.926)			
9. Desire to be a better person	−0.009	−0.025	0.047	0.070	−0.004	0.260^**^	0.044	0.059	(0.934)		
10.T-ERB	0.035	0.030	0.260^**^	0.048	0.168^**^	0.323^**^	0.157^**^	0.213^**^	0.132^**^	(0.780)	
11. Environmental knowledge	0.039	−0.053	−0.052	−0.048	−0.081	−0.472^**^	0.270^**^	0.041	0.017	−0.038	(0.836)
*M*	0.550	2.920	3.580	2.780	3.160	4.269	4.045	4.335	4.369	4.291	4.239
SD	0.499	0.827	1.301	1.006	1.395	1.382	1.755	1.546	1.645	1.243	1.399

*N* = 455, ^*^P < 0.05, ^**^P < 0.01. The diagonal brackets are Cronbach’s α.

### Hypothesis testing

We use PROCESS 3.1 to verify our hypothesis. As illustrated in Table 3, E-ERB has positively correlated with T-ERB (*β* = 0.192, *p* < 0.01), which means that H1 is supported. E-ERB is positively correlated with elevating emotions (*β* = 0.146, *p* < 0.05), views of humanity (*β* = 0.116, *p* < 0.05), and desire to be a better person (*β* = 0.308, p < 0.01). These results indicate that H2a, H2b, and H2c are supported. Both elevating emotions (*β* = 0.097, *p* < 0.05) and views of humanity (*β* = 0.085, *p* < 0.05) have a positive correlation with T-ERB, while the positive correlation between desire to be a better person and E-ERB is not supported (*β* = 0.051, *p* > 0.05), which means that H3a and H3b are supported and H3c is not supported.

**Table 3:** Results of hypotheses.

Hypotheses path	Coefficient	Standard error	*T*-value	*p*-value	Result
H1: E-ERB → T-ERB	0.192	0.040	4.775	*p* < 0.01	Supported
H2a: E-ERB → Elevating emotions	0.146	0.061	2.400	*p* < 0.05	Supported
H2b: E-ERB → Views of humanity	0.116	0.051	2.283	*p* < 0.05	Supported
H2c: E-ERB → Desire to be a better person	0.308	0.055	5.566	*p* < 0.01	Supported
H3a: Elevating emotions → T-ERB	0.097	0.030	3.249	*p* < 0.05	Supported
H3b: Views of humanity → T-ERB	0.085	0.036	2.377	*p* < 0.05	Supported
H3c: Desire to be a better person → T-ERB	0.051	0.033	1.539	*p* > 0.05	Not supported

We used bootstrapping to calculate the mediating effects (Table 4). E-ERB is positively correlated with T-ERB (effect = 0.192, SE = 0.040, 95%CI [0.113,0.271]). Elevating emotions mediate the relationship between E-ERB and T-ERB (effect = 0.014, SE = 0.007, 95%CI [0.002,0.031]). Views of humanity mediate the relationship between E-ERB and T-ERB (effect = 0.009, SE = 0.006, 95%CI [0.001,0.026]). The mediating effect of desire to be a better person is not supported (effect = 0.016, SE = 0.010, 95%CI [−0.004,0.037]). The results of the analysis of the mediating effect indicate that hypotheses H4a and H4b are supported, while hypothesis H4c is not supported.

**Table 4:** Results of mediating effect.

Path	Effect	SE	Bias-Corrected		Result
			95%CI		
			Lower	Upper	
Direct effect					
E-ERB → T-ERB	0.192	0.040	0.113	0.271	–
Indirect effects					
H4a: E-ERB → Elevating emotions → T-ERB	0.014	0.007	0.002	0.031	Supported
H4b: E-ERB → Views of humanity → T-ERB	0.009	0.006	0.001	0.026	Supported
H4c: E-ERB → Desire to be a better person → T-ERB	0.016	0.010	−0.004	0.037	Not supported

*N* = 455. SE, Standard error; CI, Confidence interval.

The results of the test for the moderating effect are specified in Table 5. Both the direct correlation between E-ERB and elevating emotions (*β* = 0.097, *p* < 0.05) and the positive relationship between E-ERB and desire to be a better person (*β* = 0.079, *p* < 0.05) are moderated by environmental knowledge. In contrast, the moderating role of environmental knowledge in E-ERB and views of humanity is not supported (*β* = 0.060, *p* > 0.05).

**Table 5:** Results of moderating effect.

Moderating variable	Path	Coefficient	Standard error	*T*-value	*P*-value
Environmental knowledge	E-ERB → Elevating emotions	0.097	0.041	2.363	*p* < 0.05
	E-ERB → Views of humanity	0.060	0.037	1.615	*p* > 0.05
	E-ERB → Desire to be a better person	0.079	0.040	1.985	*p* < 0.05

**Figure 2:**
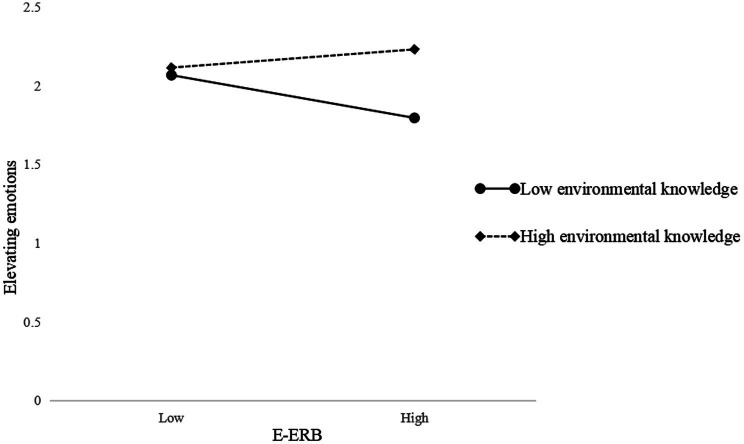
Interactive effects of E-ERB and environmental knowledge on elevating emotions.

**Figure 3:**
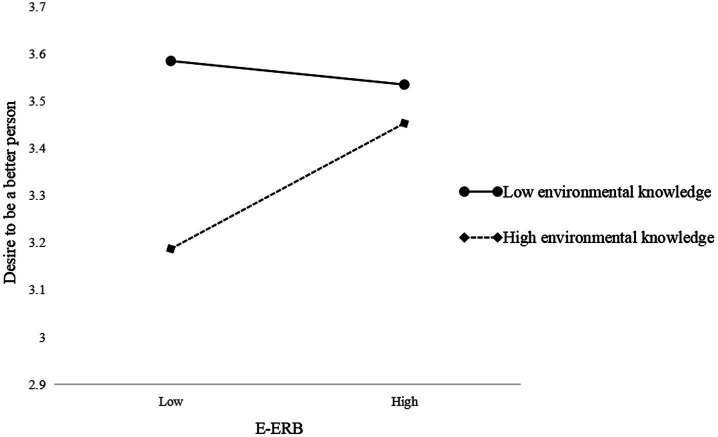
Interactive effects of E-ERB and environmental knowledge on desire to be a better person.

To visualize the moderating effect, we used Aiken and West’s (1991) method for calculating the slope (plotting the interaction with one standard deviation above and below the mean of environmental knowledge, Figures 2, 3). The effect of E-ERB on elevating emotions is amplified when environmental knowledge is high and diminished when it is low. Similarly, the effect of E-ERB on desire to be a better person is amplified when environmental knowledge is high and diminished when it is low.

According to the results of the moderated mediating effects test (Table 6), the indirect effect of E-ERB through elevating emotions on T-ERB is significant at high levels of environmental knowledge (*β* = 0.050, SE = 0.018, 95%CI [0.017, 0.089]) and similarly significant at low levels of environmental knowledge (*β* = 0.020, SE = 0.012, 95%CI [0.001, 0.047]). Thus, H5a is supported. The indirect effect of E-ERB through views of humanity on T-ERB is significant at high levels of environmental knowledge (*β* = 0.023, SE = 0.012, 95%CI [0.003, 0.050]), but not significant at low levels of environmental knowledge (*β* = 0.007, SE = 0.011, 95%CI [−0.009, 0.034]). And the indirect effect of E-ERB on T-ERB *via* desire to be a better person does not support either at high (*β* = 0.026, SE = 0.016, 95%CI [−0.006, 0.059]) or low (*β* = 0.013, SE = 0.011, 95%CI [−0.003, 0.039]) environmental knowledge. Therefore, H5b and H5c are not supported.

**Table 6:** Results of the moderated mediation effect.

Path	Moderating variable	Effect	SE	[LLCI, ULCI]	Result
H5a: E-ERB→Elevating emotions→T-ERB	High environmental knowledge(+1SD)	0.050	0.018	[0.017, 0.089]	Supported
	Low environmental knowledge(−1SD)	0.020	0.012	[0.001,0.047]	
H5b: E-ERB→Views of humanity →T-ERB	High environmental knowledge(+1SD)	0.023	0.012	[0.003, 0.050]	Not supported
	Low environmental knowledge(−1SD)	0.007	0.011	[−0.009, 0.034]	
H5c: E-ERB→Desire to be a better person →T-ERB	High environmental knowledge(+1SD)	0.026	0.016	[−0.006, 0.059]	Not supported
	Low environmental knowledge(−1SD)	0.013	0.011	[−0.003, 0.039]	

*N* = 455. SE, standard error; LLCI, lower level of confidence interval; ULCI, upper level of confidence interval.

## Discussion

Based on Haidt’s moral theory, this study established an E-ERB to T-ERB model to explain the behavioral contagion mechanism through which employees’ environmentally responsible behavior promotes tourists’ environmentally responsible behavior *via* moral elevation. Broken window theory explains the processes by which individual behavior infects groups or group norms restrict individual behavior. Nonetheless, the mechanism for inducing positive behavior in individuals needs additional investigation. Although scholars have examined the role of emotional contagion in the transmission between teachers’ sense of social responsibility and their students. And social norms theory also explains the induction of positive behavior. We believe that other mechanisms can explain behavioral contagion. Therefore, in contrast to previous studies that examined perceived destination eco-friendly reputation (Su et al., 2020), perceptions of sustainability (Lin and Lee, 2022), perceived environment quality (Liu et al., 2019), and awareness of tourism’s negative consequences (Confente and Scarpi, 2021) as environmentally responsible antecedents, this study obtains the following findings: first, T-ERB is positively influenced by E-ERB in the tourist destination. Second, only elevating emotions and views of humanity of the three moral elevation dimensions mediates the relationship between E-ERB and T-ERB. We consider that the reason why desire to be a better person does not play a mediating role may be that environmentally responsible behavior is different from the usual virtuous behavior. The beneficiaries of ordinary virtues are people or animals with living organisms. The beneficiary of an environmentally responsible act is the entire ecological environment. Moreover, the results of environmentally responsible behavior are not as intuitive as those of virtuous behavior in general. Thus, the mediating role of desire to be a better person in the relationship between E-ERB and T-ERB is not supported. Third, environmental knowledge has a moderating role in both E-ERB and elevating emotions and E-ERB and desire to be a better person. Finally, environmental knowledge moderates the indirect effects of E-ERB on T-ERB *via* elevating emotions. When environmental knowledge is high, the indirect effects of E-ERB on T-ERB *via* elevating emotions will be enhanced. In contrast, when environmental knowledge is low, the indirect effects of E-ERB on T-ERB *via* elevating emotions will be weakened.

## Theoretical contributions

First, this study expands the theoretical explanatory framework for the study of tourists’ environmentally responsible behavior. Most of the previous studies in the literature on tourists’ environmentally responsible behavior (ERB) use the theoretical frameworks of the theory of planned behavior (TPB), protection motivation theory (PMT), normative activation theory (NAM), value belief normative theory (VBN) and social norms theory to explain tourists’ ERB, without considering explanations for the contagion of individual-to-individual behavior in the tourist destination. Numerous behavioral contagion phenomena, such as the herd effect, have been identified and explained. Our study confirms the positive correlation between employees’ ERB and tourists’ ERB. This provides a new theoretical explanatory framework to explain tourists’ ERB.

Second, we examined the importance of moral elevation in enriching and expanding the study of ethical aspects within the ERB research framework. The exploration of ERB mediating variables in previous studies has focused on place identity (Lee and Oh, 2018), environmental attitudes (Safshekan et al., 2020), place attachment (Lin and Lee, 2022), and subjective norm (Liu et al., 2019). Although many theories argue that ethics is a major predictor variable of individual behavior, only a few scholars have added environmental ethics (Zheng and Dai, 2012) to the framework of ERB research. Moral elevation can be achieved through the positive and pleasurable emotions triggered by witnessing the ethical behavior of others and accompanied by the desire to imitate. We propose and validate the process by which tourists generate elevating emotions and thus influence their ERB by witnessing their employees’ ERB. This provides new insights for future research frameworks exploring the role of moral factors in ERB. In addition, we expanded the boundary of the T-ERB research by including environmental knowledge as a moderating variable between the E-ERB and moral elevation. The main moderating variables of previous studies on ERB are environmental sensitivity, place attachment, and the new ecological paradigm. We expanded the examination of ERB’s boundary.

## Management implications

Although the rules and regulations of the scenic spot will compulsorily regulate the uncivilized behavior of tourists, they cannot stimulate the active behavior of tourists to protect the tourist destination. Our study used Haidt’s moral elevation theory to explain the contagion mechanism between employees’ behavior and tourists’ behavior. Our study revealed that the impact of the ERB of employees in tourist destinations on the ERB of tourists is achieved by increasing their moral enhancement. Therefore, we suggest that managers of tourist destinations can increase the ERB of tourists in their tours through additional behaviors that increase their moral elevation, such as inviting tourists to submit their photographs of employees engaging in environmentally responsible behavior and display them at the destination. Meanwhile, some volunteers were recruited from employees to use their free time to participate in environmental protection projects in tourist destinations. According to Haidt’s experiment, these behaviors can increase tourists’ moral elevation.

Our study confirms the influence of environmental knowledge on the ERB through the moral improvement of tourists through employees’ ERB. Many studies suggest that as individuals’ environmental knowledge increases, more ecological behaviors occur (Ogbeide et al., 2015). Therefore, environmental knowledge should be enhanced for both employees and tourists. Through environmental legislation, the government can encourage tourism companies to pay attention to environmental issues and learn about the environment in tourist destinations. According to the downside impact, the employee’s environmental knowledge will increase, and therefore, more ERB will appear in tourism destinations. On the other hand, there are many sources of environmental knowledge for tourists. The government or tourism enterprises can strengthen tourists’ environmental knowledge through self-media propaganda, whereas tourist destinations can strengthen tourists’ environmental knowledge by occasionally playing environmental knowledge to inspire them and by coordinating environmental knowledge quiz activities.

## Limitations and recommendations for future research

This study has several limitations that present future research opportunities. The data used to estimate E-ERB were collected from tourists. Individuals’ observations of others’ ERB may vary due to personality variables, and these observations may be inaccurate. Future studies should use longitudinal data or causal experiments to clarify the findings made in this study. We believe there are numerous types of destination employees, and some employees’ environmentally responsible behaviors are viewed as part of their job responsibilities (e.g., sanitation workers), while others are not (e.g., managers, office staff). We suggest that these types can be distinguished and included in future studies. Further, we collected data twice for the same questionnaire. This may have prompted participants to become sidetracked by other variables in the questionnaire or lose patience due to the questionnaire’s excessive length. These can compromise the accuracy of the data. Future surveys should collect information on different variables at two or three separate times to assure data accuracy. Finally, our research is based on Haidt’s idea of moral enhancement, which suggests that the moral behavior of employees encourages the moral behavior of tourists. In future research, we propose using different theoretical models, such as the herding effect, to investigate the motivating approach of T-ERB.

## Conclusion

This study focuses on the impact of employees’ environmentally responsible behavior in tourist destinations on tourists’ environmentally responsible behavior. We verified the important role of moral elevation in the transfer of environmentally responsible behavior of employees and tourists based on Haidt’s moral perspective. We propose a new perspective to explain the transmission mechanism between employees’ environmentally responsible behavior and tourists’ environmentally responsible behavior in tourism destinations, which will help to expand our understanding of the relationship between employees’ behavior and tourists’ behavior. Simultaneously, we provide new supporting evidence for the study of behavioral contagion. Besides, by incorporating environmental knowledge as a moderating variable in our model, we further broaden the scope of the research on environmentally responsible behavior. We expect our study to spark more exploration of the contagion of positive behavior in the field of environmental psychology.

## Data availability statement

The original contributions presented in the study are included in the article/Supplementary material, further inquiries can be directed to the corresponding author.

## Author contributions

WY wrote the first draft and collected the data. ZL processed the data and revised the manuscript. YX participated in the data collection. All authors contributed to the article and approved the submitted version.

## Funding

This work was funded by research on the development path of powerful agricultural specialties towns in the background of rural revitalization (Project commissioned by Fujian Provincial Department of Finance, KLE21002A).

## Conflict of interest

The authors declare that the research was conducted in the absence of any commercial or financial relationships that could be construed as a potential conflict of interest.

## Publisher’s note

All claims expressed in this article are solely those of the authors and do not necessarily represent those of their affiliated organizations, or those of the publisher, the editors and the reviewers. Any product that may be evaluated in this article, or claim that may be made by its manufacturer, is not guaranteed or endorsed by the publisher.
