# Supplementary material for: Transmission of environmentally responsible behavior between tourist destination employees and tourists: The role of moral elevation and environmental knowledge
Source: Front Psychol. 2022 Nov 30;13:1027736. doi: 10.3389/fpsyg.2022.1027736 (PMC9748094; doi:10.3389/fpsyg.2022.1027736)
Supplement: Supplementary file 1 [file Data_Sheet_1.docx]

**Appendix 1**

The measure items used in this study

| **Employees’ environmentally responsible behavior** |
| --- |
| 1. Employees of tourist destinations take the initiative to stop others from being destructive to the environment of (name of destination). 2. When tourist destination employees saw others engaged in the destruction of the environment at (name of destination), he/she will stop it and report it to relevant units immediately. 3. When the employee of tourist destinations saw garbage and tree branches on the ground, he/she will pick them up and put them in the trash. 4. Employees of tourist destinations take the initiative to attend environmental clean-up activities at (name of destination). 5. Employees of tourist destinations convince others to protect the natural environment at (name of destination). 6. Employees of tourist destinations never disrupt the fauna and ﬂora at (name of destination). |
| **Tourists’ environmentally responsible behavior**   1. I follow the legal ways to stop the destruction of the environment of (name of destination) 2. When I see others engaged in the destruction of the environment at (name of destination), I will report it to the destination administration or relevant units. 3. When I see garbage and tree branches on the ground, I will pick them up and put them in the trash. 4. If there are environmental clean-up activities at (name of destination), I would be willing to attend. 5. I try to convince others to protect the natural environment at (name of destination). 6. I try not to disrupt the fauna and ﬂora during my travel. |
| **Moral Elevation** |
| Elevating emotions   1. The proactive behaviors of the tourist destination’s employees to protect the environment makes me feel moved. 2. The proactive behaviors of the tourist destination’s employees to protect the environment makes me feel admiration. 3. The proactive behaviors of the tourist destination’s employees to protect the environment makes me feel inspired. |
| Views of humanity   1. The proactive behaviors of the tourist destination’s employees to protect the environment makes me believe that there is still some good in the world. 2. The proactive behaviors of the tourist destination’s employees to protect the environment makes me believe that the world is full of kindness and generosity. 3. The proactive behaviors of the tourist destination’s employees to protect the environment makes me believe that people are really good. 4. The proactive behaviors of the tourist destination’s employees to protect the environment makes me believe that there are still a few good people out there. |
| Desire to be a better person   1. The proactive behaviors of the tourist destination’s employees to protect the environment makes me desire to be a better person. 2. The proactive behaviors of the tourist destination’s employees to protect the environment shows me how to be a better person. 3. The proactive behaviors of the tourist destination’s employees to protect the environment makes me desire to do more to protect the environment. 4. The proactive behaviors of the tourist destination’s employees to protect the environment makes me desire to participate in protecting the environment. |
| **Environmental knowledge**   1. Carbon dioxide contributes to the creation of the greenhouse effect. 2. Overusing fertilizer and pesticide will damage the environment. 3. Using detergent powder containing phosphorus will not cause water pollution. 4. Chloro ﬂuoro carbons (CFC) emission from refrigerators is one of the causes to ozone depletion 5. Acid rain has nothing to do with coal burning. 6. Due to the interdependency among species, one species vanishing will cause chain reactions. 7. The increasing carbon dioxide in the atmosphere is one of the factors causing a warming climate. |
